# Learning from an Emerging Infection: How the COVID-19 Pandemic Reshaped Gastric Cancer Care

**DOI:** 10.3390/life16010161

**Published:** 2026-01-19

**Authors:** Alexandru Marian Vieru, Dumitru Radulescu, Liliana Streba, Emil Tiberius Trasca, Sergiu Marian Cazacu, Razvan-Cristian Statie, Petrica Popa, Tudorel Ciurea

**Affiliations:** 1 Doctoral School, University of Medicine and Pharmacy of Craiova, Petru Rares Street No 2-4, 200349 Craiova, Romania; 2 General Surgery Department, University of Medicine and Pharmacy of Craiova, Petru Rares Street No 2-4, 200349 Craiova, Romania; 3 Oncology Department, University of Medicine and Pharmacy of Craiova, Petru Rares Street No 2-4, 200349 Craiova, Romania; 4 Gastroenterology Department, University of Medicine and Pharmacy of Craiova, Petru Rares Street No 2-4, 200349 Craiova, Romania

**Keywords:** gastric cancer, COVID-19, early diagnosis, endoscopy, therapeutic strategies, vaccination, telemedicine, healthcare resilience

## Abstract

**Background/Objectives:** The COVID-19 pandemic profoundly disrupted gastric cancer care, reducing access to screening, delaying diagnosis, and altering therapeutic pathways worldwide. Beyond clinical challenges, it exposed structural weaknesses in healthcare systems but also accelerated innovation. **Methods:** We conducted a narrative review supported by a structured literature search (PubMed/MEDLINE, Scopus, Web of Science; 1 January 2014–30 November 2025), with a narrative synthesis of observational studies, registry analyses, and meta-analyses addressing COVID-19–related changes in gastric cancer epidemiology, diagnosis, treatment, vaccination, and telemedicine. A PRISMA-style flow diagram was used to illustrate study selection. **Results:** Elective endoscopy volumes fell by up to 80%, leading to diagnostic backlogs and increased proportions of advanced-stage gastric cancer. Surgical postponements, modified chemotherapy and radiotherapy schedules, and reduced molecular/genetic testing further compromised outcomes. Conversely, vaccination, telemedicine, capsule endoscopy, and adaptive triage frameworks enabled partial recovery of services. Geographical variations were observed in the recovery of gastric cancer care services, with regions that had established screening infrastructure generally resuming activity more rapidly, whereas others experienced ongoing delays and diagnostic backlogs. **Conclusions:** This review integrates epidemiological, diagnostic, and therapeutic evidence to demonstrate how COVID-19 redefined gastric cancer care. By highlighting regional disparities and outlining a conceptual model for oncologic resilience, it provides an innovative framework for future crisis preparedness. The lessons of the pandemic—digital health integration, flexible treatment protocols, and international collaboration—represent a foundation for more robust, equitable gastric cancer management in the post-pandemic era.

## 1. Introduction

### 1.1. Global Context of Gastric Cancer

Gastric cancer represents a major public health challenge worldwide, with over one million new cases each year and remaining one of the leading causes of cancer-related deaths despite a downward trend in recent decades [1,2].

This decline is largely attributed to improved socioeconomic conditions, enhanced hygiene practices, and better food-preservation techniques, particularly in developed regions; however, the overall number of cases remains high due to population aging and ongoing demographic growth [3].

Geographically, the incidence is notably high in East Asia; according to recent GLOBOCAN estimates (2022), age-standardized incidence rates (ASR, world standard) are 13.7 per 100,000 in China and approximately 27 per 100,000 in Japan (27.6) and South Korea (27.0), supporting the substantial regional burden and associated mortality impact [4]. In countries such as Japan and South Korea, although the absolute number of cases is still considerable, the implementation of population-based endoscopic screening programs has led to more frequent early-stage diagnoses and improved survival [5,6].

By comparison, the incidence is lower in Western Europe and North America, with age-standardized incidence rates (ASR, world standard) of 6.4 per 100,000 in Germany and 4.1 per 100,000 in the United States (GLOBOCAN 2022). In these regions, a relative shift toward proximal/cardia tumors has been described, and proximal (cardia) gastric adenocarcinoma is linked to obesity and gastroesophageal reflux disease [4,7,8].

In resource-limited regions like parts of Africa and Latin America, inadequate epidemiological data combined with poor access to healthcare continue to contribute to high mortality rates [9,10].

*Helicobacter pylori* infection remains the primary etiological factor, accounting for the vast majority of non-cardia gastric cancers (≈89%) and a substantial share of overall gastric cancers globally [11].

The bacterium induces chronic inflammation and atrophic gastritis, and when coupled with smoking and a high-salt, preserved-food diet, it can trigger preneoplastic changes that progress to cancer [12,13,14].

Genetic predisposition also plays a role, ranging from well-defined familial syndromes (such as CDH1 mutations) to less specific family history, while dietary patterns—particularly high consumption of processed foods and low intake of fruits and vegetables—further contribute to risk [12,14,15].

Despite advances in screening and treatment, the global burden of gastric cancer remains substantial; therefore, prevention strategies—risk-factor reduction, *Helicobacter pylori* eradication, early diagnosis, and modern therapies—are critical for lowering mortality [2,3,11,12,13,14,15].

### 1.2. The Role of Upper Gastrointestinal Endoscopy in Gastric Cancer Diagnosis

Upper gastrointestinal endoscopy is the preferred method for detecting and characterizing gastric lesions with malignant potential, as it permits direct visualization of the mucosa and enables targeted biopsies of suspicious areas [16,17].

In countries like Japan and South Korea, the integration of endoscopy into national screening programs has resulted in the frequent detection of early-stage gastric cancer, which directly improves survival outcomes [5,6].

Compared to barium radiography, endoscopy offers superior sensitivity for detecting early lesions, with the added advantage of providing tissue samples for histopathological confirmation [18].

The use of advanced imaging modalities, such as narrow-band imaging and magnifying endoscopy, further enhances visualization of subtle mucosal changes and allows more precise characterization of suspicious lesions [19].

The quality of the examination and the expertise of the endoscopist are critical factors in ensuring high detection rates for early gastric lesions, and repeat endoscopies in patients with preneoplastic conditions or under surveillance can increase the likelihood of detecting progression toward advanced disease [16,18].

Beyond diagnosis, endoscopy also facilitates minimally invasive therapeutic interventions such as endoscopic mucosal resection (EMR) and endoscopic submucosal dissection (ESD). These techniques enable en bloc removal of early lesions, which is essential for accurate histological assessment and for minimizing local recurrence [20,21,22,23].

Thus, upper gastrointestinal endoscopy represents a cornerstone in reducing gastric cancer mortality, spanning the continuum from screening and diagnosis to early therapeutic management.

### 1.3. Objective of the Narrative Review

This review aims to assess the impact of the COVID-19 pandemic on the diagnosis and management of gastric cancer by examining epidemiological trends and the practical consequences of mobility restrictions and healthcare system reorganization.

It highlights how reduced access to endoscopic investigations and disruptions in treatment flows have led to delays in early diagnosis and an increased proportion of advanced-stage cases.

Additionally, the review explores adaptations in chemotherapy, radiotherapy, and surgical protocols for gastric cancer patients, emphasizing the roles of vaccination and telemedicine in restoring optimal oncologic services.

Finally, it discusses future directions, including enhanced screening and *Helicobacter pylori* eradication efforts, the development of adaptable emergency plans, and the importance of international collaboration to ensure the continuity of patient care during global crises.

While previous literature has addressed the general impact of the COVID-19 pandemic on oncology and, in some instances, on gastric cancer specifically, there is a lack of comprehensive reviews that systematically integrate epidemiological data, practical diagnostic and therapeutic challenges, and adaptive strategies developed during the pandemic. This review distinguishes itself by:

• Providing an integrated analysis of epidemiological, diagnostic, and therapeutic changes in gastric cancer care during the COVID-19 pandemic;
• Comparing the impact and recovery strategies across regions with different healthcare resources;
• Identifying current knowledge gaps and proposing a conceptual framework for future crisis management in gastrointestinal oncology.

**Figure 1:**
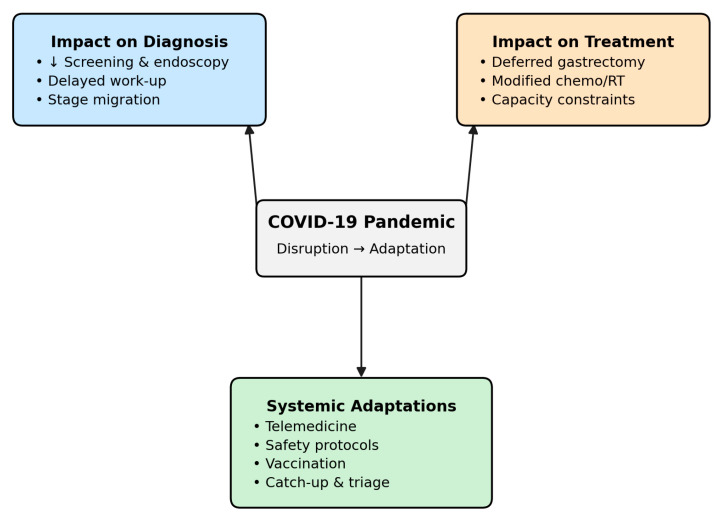
Conceptual framework of the impact of COVID-19 on gastric cancer care. Arrows indicate the directional influence of the COVID-19 pandemic on diagnostic and treatment pathways, and on systemic adaptations.

By adopting this approach, the present review offers both a critical synthesis of the available evidence and practical recommendations, aiming to inform clinical practice and guide future research in the field. An integrative overview of these objectives is summarized in Figure 1, which illustrates the conceptual framework of pandemic-related disruptions and adaptive strategies in gastric cancer care.

## 2. Materials and Methods

### 2.1. Design of the Review

**Figure 2:**
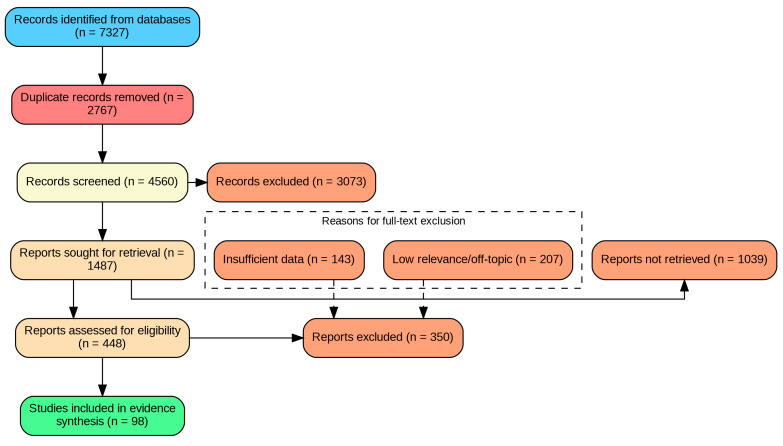
PRISMA flow diagram of the study selection process.

This study is a narrative review supported by a structured literature search, designed to synthesize and contextualize the available evidence regarding the impact of the COVID-19 pandemic on gastric cancer care. Given the use of explicit eligibility criteria and a transparent study selection process, a PRISMA-style flow diagram was used to illustrate article identification, screening, and inclusion (Figure 2). A completed PRISMA 2020 checklist, used as a reporting and transparency aid for the selection process, is provided as Supplementary File S1. No meta-analysis was planned or performed due to substantial heterogeneity across study designs and outcomes.

### 2.2. Information Sources and Timeframe

The bibliographic search was performed in three major databases: PubMed/MEDLINE, Scopus, and Web of Science, covering publications from 1 January 2014 to 30 November 2025. Only full-text, peer-reviewed articles published in English and involving human subjects were considered. The inclusion of pre-pandemic studies (2014–2019) allowed the establishment of a baseline for epidemiological trends and management practices, providing context for the analysis of pandemic-related disruptions.

### 2.3. Search Strategy

Search queries combined controlled vocabulary and free-text terms related to gastric cancer and pandemic.

For PubMed/MEDLINE, the core search string was:

(“Stomach Neoplasms”[Mesh] OR “gastric cancer” OR “stomach cancer”) AND (“COVID-19”[Mesh] OR “SARS-CoV-2” OR “coronavirus disease 2019” OR pandemic) AND (screening OR endoscopy OR diagnosis OR surgery OR chemotherapy OR radiotherapy OR telemedicine OR vaccination).

Equivalent strategies, adapted to database-specific syntax and subject headings, were used in Scopus and Web of Science, searching titles, abstracts, and keywords. No study-design filters were applied at the search stage, in order to maximize sensitivity. Keyword selection was guided by expert consensus among authors and an initial exploratory screening to ensure comprehensive coverage of epidemiology, diagnosis, treatment, vaccination, and telemedicine.

### 2.4. Eligibility Criteria

Inclusion: Observational studies, registry analyses, clinical series, and meta-analyses reporting data on incidence, stage distribution, diagnostic pathways (including endoscopy), treatment adaptations (surgery, systemic therapy, radiotherapy), or pandemic-specific interventions (telemedicine, vaccination).

Exclusion: Reports without clear study periods, those focusing exclusively on non-gastric neoplasms, or studies with small/ambiguous cohorts lacking interpretable outcomes.

Guidelines/consensus statements, editorials/letters, and methodological reports were included as contextual/background sources to describe practice changes, but were not treated as core evidence for quantitative statements.

### 2.5. Study Selection Process

The initial search yielded 7327 records. All records were exported to a reference manager and duplicates were removed prior to screening. Titles/abstracts and full texts were screened independently by two reviewers, with disagreements resolved by consensus. After screening, 1487 reports were sought for retrieval; 1039 could not be retrieved in full text. The remaining 448 reports were assessed for eligibility. Following full-text assessment, 350 reports were excluded (207 for low relevance/off-topic and 143 for insufficient data), resulting in 98 sources included in the evidence synthesis. The study selection process is summarized in the PRISMA flow diagram (Figure 2).

### 2.6. Study Typology

**Figure 3:**
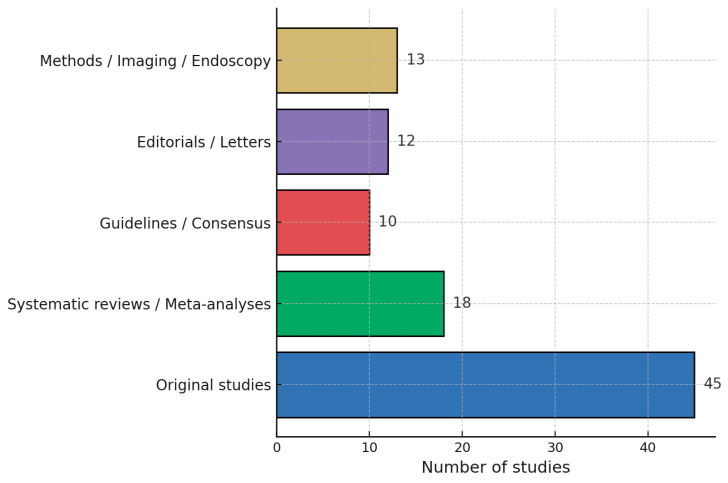
Distribution of the 98 Included Studies. Original studies: 45. Systematic reviews/Meta-analyses: 18. Guidelines/Consensus statements: 10. Editorials/Letters: 12. Methodological/Imaging/Endoscopy reports: 13.

The 98 included sources were heterogeneous in methodology. Their distribution by type is summarized in Figure 3—study typology:

### 2.7. Data Extraction and Synthesis

From each study we extracted design, setting, sample size, time period, key outcomes (incidence, staging, diagnostic or treatment delays, outcomes), and pandemic-related adaptations (triage, telemedicine, vaccination strategies). Data were extracted using a standardized form developed a priori; extraction was cross-checked, and any discrepancies were resolved by consensus (inter-rater statistics were not calculated). Given heterogeneity, a narrative synthesis was performed rather than a meta-analysis.

### 2.8. Quality and Risk of Bias Assessment

Quality appraisal was performed using criteria adapted from Joanna Briggs Institute (JBI) tools for observational research. Risk-of-bias appraisal was applied only to data-driven empirical studies (observational studies/registry analyses/clinical series and meta-analyses). Non-empirical sources (guidelines, editorials/letters, methodological reports) were not formally appraised and were used for context only.

Empirical studies providing original data (e.g., observational studies, clinical series, registry/claims analyses) were included in the core evidence synthesis and underwent quality/risk-of-bias assessment. Editorials, letters, and methodological/technical reports were included only as contextual/background sources to describe practice adaptations and emerging concepts; these were not subject to formal risk-of-bias appraisal and were not used as sole support for main conclusions. Non-empirical sources and background references were used for context and framing; therefore, the total number of references (*n* = 107) exceeds the number of sources included in the evidence synthesis (*n* = 98).

In practice, high-risk findings were not used as standalone support for key conclusions; they were reported descriptively and interpreted in light of larger/more robust datasets.

## 3. The COVID-19 Pandemic and the Stomach: Effects and Potential Mechanisms

### 3.1. Interaction Between SARS-CoV-2 and the Gastrointestinal Tract

Since the early stages of the pandemic, SARS-CoV-2 has been shown to interact with the gastrointestinal tract, supported by ACE2 receptor expression on gastric and intestinal epithelial cells and by reports of viral RNA/particles in mucosal tissue [24,25,26,27]. However, in the context of gastric oncopathology, the clinical relevance of these observations appears mainly indirect and remains incompletely defined.

Available data suggest that COVID-19 may be associated with transient mucosal injury, systemic inflammation, and (in some cohorts) gut dysbiosis and increased intestinal permeability [25,28,29,30,31,32]. These pathways have been proposed as potential contributors to persistent gastrointestinal symptoms and to inflammatory changes that could, in theory, influence pre-existing high-risk gastric conditions [33,34,35,36,37]. Importantly, the long-term impact on gastric premalignant lesions and carcinogenesis remains largely hypothetical, and current evidence does not demonstrate a direct or accelerated pathway of gastric cancer development attributable to SARS-CoV-2 [28,33,34].

From a practical standpoint, the overlap between nonspecific gastrointestinal symptoms during COVID-19 and symptoms that may also occur in gastric cancer may complicate clinical assessment and contribute to delayed investigation—particularly when routine diagnostic services are disrupted [29,33].

### 3.2. COVID-19 and Gastric Pathology

The COVID-19 pandemic introduced several potential biological and behavioral factors that may influence preexisting gastric conditions; however, the long-term relevance of these mechanisms to gastric carcinogenesis remains uncertain. Reports have linked COVID-19–related systemic inflammation, hypoxia, coagulopathy, and medication exposure (e.g., corticosteroids/anti-inflammatory drugs) with mucosal injury and exacerbation of ulcer-related complications, while psychological distress and lifestyle changes during lockdowns were associated with worsening gastrointestinal symptom burden in patients with functional and organic disorders [35,36,37,38,39,40,41,42].

Importantly, hypotheses suggesting that persistent inflammation or immune dysregulation after SARS-CoV-2 infection could accelerate progression of premalignant lesions (especially in the presence of *Helicobacter pylori*) remain speculative, and current evidence does not establish a direct causal link [43,44,45]. Therefore, in the context of gastric cancer, the most consistent and actionable impact of the pandemic is mediated through disruptions in clinical pathways and healthcare delivery, as summarized below.

Clinically, the pandemic’s impact on gastric disease care has been driven primarily by disruptions in clinical pathways, including reduced diagnostic endoscopy capacity, delayed evaluation, and altered referral patterns [46,47,48,49,50,51]. For example, in South Korea, the annual number of esophagogastroduodenoscopies decreased by 6.3% in 2020 compared with 2019, and claims for advanced gastric cancer decreased by 3.6% [50]. In parallel, the gastric cancer screening rate decreased from 70.8% (2019) to 68.9% (2020), while screening within the past year dropped from 32.7% to 27.2% [51]. These healthcare-delivery effects and their downstream implications for stage at diagnosis and treatment delivery are addressed in detail in the dedicated sections on diagnostic disruption and therapeutic management.

## 4. COVID-19 and Gastric Cancer: Incidence and Risk Factors

### 4.1. Epidemiological Changes

The COVID-19 pandemic period was associated with a higher proportion of late-stage gastric cancer diagnoses, likely reflecting disruptions in screening pathways and reduced access to elective endoscopy services [52,53,54].

For example, in Hiroshima (Japan), early-stage gastric cancer diagnoses decreased by more than 20% during the early COVID-19 period (e.g., stage I −23.9%) [52]. Similarly, a large single-institute cohort from Yonsei Cancer Center (South Korea) reported fewer diagnoses in 2020–2021 than in 2018–2019 (6336 vs. 4539; 264 vs. 189 cases/month; −28.4%), alongside a higher proportion of advanced-stage disease particularly in 2021 (stage IV and stage III–IV) [55]. A study-level summary of the main numerical signals of diagnostic disruption and stage migration is provided in Table 1.

Similar trends were observed in Italy and other European countries, where diagnostic rates dropped by as much as 15.9%, and in the United States, with an estimated annual decline of approximately −8.9% [46,48].

In resource-limited settings, such as parts of the Middle East and South Asia, delays in gastric cancer diagnosis were also reported due to healthcare system disruptions and reduced access to endoscopy, although specific national data remain limited [38,47].

Across multiple regions, temporary reductions in diagnostic activity and delays in referral were associated with fewer early detections and a higher proportion of locally advanced or metastatic presentations [38,46,54]. Patient hesitancy to seek medical care during surges likely compounded these effects.

Five-year survival rates for gastric cancer exceed 90% in stage I, drop to approximately 30% in stage III, and fall below 10%—with some analyses reporting rates under 3%—in stage IV, according to large-scale cohort studies and survival meta-analyses [56,57].

Moreover, the pandemic has revealed significant geographical disparities in gastric cancer care. In countries with established screening infrastructure—such as South Korea and certain regions of Japan—diagnostic volumes recovered more rapidly, which helped offset some negative outcomes [50,52]. In contrast, countries without centralized national screening policies or with underfunded public healthcare systems, including parts of Eastern Europe, Latin America, and the Middle East, experienced prolonged service disruptions and persistent diagnostic backlogs [38,46,54]. For example, South Korea saw a near-complete recovery to pre-pandemic gastric cancer screening rates within one year, whereas resource-limited settings—such as many countries in Africa—continued to experience substantial diagnostic backlogs and service disruptions months after initial pandemic waves [50]. These disparities are further exacerbated by socioeconomic inequality and unequal access to high-volume cancer centers, highlighting the need for targeted public health interventions and investment in screening programs to reduce the risk of increased gastric cancer mortality globally.

### 4.2. Specific Risk Factors in the Pandemic Context

Beyond disrupting healthcare systems, the pandemic has also reshaped population behaviors related to lifestyle and adherence to preventive medical care, thereby influencing risk factors associated with gastric cancer. Fears of hospital-acquired infections and hospitalization significantly hindered early diagnostic efforts. Multiple surveys and observational studies reported that patients were frequently reluctant to undergo endoscopic evaluations, even when presenting with alarming gastrointestinal symptoms—leading to delayed presentations and more advanced disease at diagnosis [37,58,59].

Additionally, lifestyle changes during lockdowns may have increased population-level vulnerability to cancer risk factors. The closure of fitness facilities and limits on outdoor mobility promoted sedentary behavior, while pandemic-related stress was frequently associated with higher consumption of ultra-processed foods rich in salt, sugar, and saturated fats [60].

These behavioral shifts are known to contribute to obesity and low-grade systemic inflammation—both established risk factors for gastric and other gastrointestinal cancers [15,61].

Simultaneously, collective protective measures have redirected medical equipment and staff toward COVID-19 emergencies, causing delays in testing for and eradicating *Helicobacter pylori*. For many patients, either H. pylori testing has been postponed or eradication therapies have been interrupted or shortened, potentially favoring the persistence of chronic gastritis and the progression of preneoplastic lesions in the long term [62].

Furthermore, although genetic predisposition plays a key role in gastric cancer risk, genetic counseling and molecular testing pathways were interrupted in many oncology centers due to lockdowns, staff shortages, and prioritization of urgent care. Genetic services experienced measurable declines during the first lockdown waves. For instance, at a centralized molecular diagnostics center in Serbia, overall molecular analyses fell by 38% during the state of emergency, and only 3 of 48 scheduled pre-test genetic counseling appointments were conducted; in parallel, somatic BRCA1/2 testing was temporarily suspended [63]. Similarly, a large tertiary cancer genetics clinic reported a decrease in referral volume (median 35 vs. 22 referrals/week; *p* < 0.001) and reduced genetic testing uptake (97.7% [176/180] vs. 74.1% [180/243]; *p* < 0.001), with the median time from pre-test counseling to blood draw increasing from 0 to 11 days (*p* < 0.001) [64].

These disruptions decreased access to timely risk assessments and preventive strategies, raising concerns that individuals with hereditary cancer syndromes may have missed early interventions and later received diagnoses at more advanced stages. In this way, the pandemic has not only altered healthcare delivery but also introduced new challenges in managing the global risk factors for gastric cancer—from dietary habits and lifestyle to H. pylori eradication and the proper follow-up of individuals with hereditary predispositions.

## 5. Gastric Cancer in COVID-19 Positive Patients

### 5.1. Association of COVID-19 Infection with the Progression of Gastric Cancer

The occurrence of COVID-19 infection in patients with gastric cancer has been a significant concern since the early reports on the pandemic’s impact on oncology populations. In gastric cancer, tumor-induced immunosuppression—along with the clinical fragility often associated with advanced disease and multiple comorbidities—can significantly worsen the severity of SARS-CoV-2 infection, increasing the risk of respiratory failure and systemic complications [65,66].

Studies have shown that gastric cancer patients infected with COVID-19 face higher hospitalization and mortality rates compared to cancer-free individuals, with mortality estimates ranging from 20% to over 35%, depending on stage and treatment status [65,66,67].

Unlike many other malignancies, gastric cancers are often accompanied by poor nutritional status, with patients frequently presenting with anemia and hypoalbuminemia, conditions that further impair the body’s ability to withstand severe infections [39,40].

An emerging hypothesis suggests that the interplay between chronic inflammation within the tumor microenvironment and the cytokine storm induced by SARS-CoV-2 infection may exacerbate immune dysfunction in oncology patients. This theoretical synergy could further impair antitumor immunity and contribute to disease progression [68,69].

Additionally, systemic cancer treatments (such as chemotherapy or targeted therapies) are known to suppress immune responses, potentially rendering SARS-CoV-2 infections more aggressive and necessitating intensified clinical monitoring [68].

Systemic hypoxia and coagulopathy seen in severe COVID-19 cases can also worsen outcomes, predisposing patients—particularly those with malignancies—to thromboembolic events such as deep vein thrombosis and pulmonary embolism [41]. Such individuals are highly susceptible to deep vein thrombosis and pulmonary embolism due to overlapping mechanisms of cancer-associated thrombosis (CAT) and COVID-associated coagulopathy (CAC), and these thrombotic complications contribute significantly to the elevated mortality observed in cancer patients with COVID-19 [41,65].

Clinically, patients with gastric cancer who contract COVID-19 face an increased risk of hemodynamic instability and acute complications such as gastrointestinal hemorrhage or ulcer perforation related to the neoplasm. This risk is compounded by recent oncologic therapies, which may deplete hematologic reserves and heighten systemic toxicity. Moreover, evolving hospitalization protocols and the need for repeated SARS-CoV-2 testing can further delay essential diagnostic or therapeutic interventions [47,49].

### 5.2. Diagnostic and Therapeutic Considerations

The COVID-19 pandemic has directly affected the capacity to diagnose and treat oncology patients, with this impact particularly pronounced among gastric cancer patients with active SARS-CoV-2 infection. In many centers, both endoscopic and imaging investigations have been subject to triage based on urgency, often resulting in postponed or canceled staging procedures for patients who tested positive [47,70].

To mitigate risk and manage limited resources, some institutions implemented formal triage frameworks prioritizing patients based on tumor aggressiveness, COVID-19 status, and overall prognosis [71,72]. Moreover, certain imaging modalities—such as contrast-enhanced computed tomography—were temporarily limited mainly due to staff shortages, workflow constraints, and the redirection of hospital resources toward intensive care, with infection-control considerations also contributing.

Surgical interventions (including partial or total gastrectomies) in COVID-19 positive patients carry an increased risk for both the patient and the medical team, given the aerosol-generating nature of general anesthesia and the inherent complexity of these procedures [49]. Consequently, strict preoperative testing and isolation protocols have been adopted, with surgeries often deferred until the patient recovers or achieves a negative virological status—provided the oncologic stage allows for such a delay. In cases of rapid tumor progression or complications such as acute bleeding, perforation, or obstruction, urgent surgical interventions have been carried out under enhanced protective measures and COVID-19-specific protocols [49]. For example, institutions in Italy and Iran reported increases in emergency gastric surgeries, such as subtotal gastrectomies and bleeding ulcer repairs, performed in negative-pressure operating rooms with full PPE and reduced staff to minimize exposure risk [46,53].

Chemotherapy and immunotherapy regimens have also undergone adjustments. For gastric cancer patients with active SARS-CoV-2 infection, treatment cycles are generally delayed or rescheduled until post-infection recovery to avoid the compounded risks of severe toxicity and immunosuppression [73,74]. The use of hypofractionated radiotherapy and oral treatment regimens has been employed as strategies to minimize hospital visits and reduce exposure to high-risk environments. Despite these measures, there remains concern that such delays may result in missed therapeutic opportunities—such as failure to initiate systemic chemotherapy within the optimal time window or the inability to perform timely surgical resection due to incomplete staging—particularly in patients with advanced disease who depend on coordinated, prompt interventions for curative or palliative outcomes [74,75].

In this challenging context, multidisciplinary teams—comprising oncology, gastroenterology, surgery, and radiotherapy—have been critical in establishing optimal management strategies, all while adhering to stringent safety protocols for healthcare providers and other patients. The inherently volatile nature of the pandemic, marked by successive waves and evolving restrictions, has necessitated continual adaptation of therapeutic plans and transparent communication with patients regarding the risks and benefits of each treatment option [37,76].

## 6. Gastric Cancer During the COVID-19 Pandemic

### 6.1. Incidence of Endoscopic Procedures and Delayed Diagnosis

The restrictions imposed by the COVID-19 pandemic were associated with a marked reduction in endoscopic procedures—both screening and diagnostic—which may have hampered opportunities for early detection of gastric cancer. Many centers experienced a 60–80% drop in elective endoscopies during the pandemic’s peak months, a consequence of both the diversion of resources to treat SARS-CoV-2-infected patients and widespread patient apprehension about potential hospital-acquired infections [47,48,49]. In the United Kingdom, analysis of the National Endoscopy Database (NED) demonstrated that endoscopic activity fell to just 5% of pre-pandemic levels at the nadir, before rebounding to approximately 20% by May 2020 following targeted vetting and catch-up initiatives [58]. In the United States, data from the GI Quality Improvement Consortium (GIQuIC) registry revealed a 33.4% decrease in esophagogastroduodenoscopy (EGD) and a 38.5% decrease in colonoscopy volumes between March and September 2020, which guided the development of structured recovery plans [59]. Consequently, a substantial number of patients with early symptoms of gastric cancer have delayed seeking medical advice, often presenting at more advanced stages when clinical manifestations are more severe and treatment options are more limited.

This decline in routine investigations has created a diagnostic gap for preneoplastic or early gastric lesions, potentially leading to a higher number of cases with locoregional spread or metastases at the time of diagnosis [43,44]. The reduction in routine endoscopies has also limited opportunities for minimally invasive procedures such as EMR or ESD—interventions that are most effective when the disease is caught early. If these screening deficits are not addressed through recovery strategies, the long-term effect could be an increased mortality rate associated with gastric cancer [54].

### 6.2. Relationship Between Preneoplastic and Neoplastic Lesions

During the pandemic, the underdiagnosis of atrophic gastritis and intestinal metaplasia has raised significant concerns among gastroenterologists. Without regular consultations and periodic endoscopic surveillance, these preneoplastic conditions may progress over time to dysplasia and eventually to gastric adenocarcinoma, depriving patients of the opportunity for preventive management [43,44]. The constraints on endoscopic procedures have left many high-risk cases undetected, and cumulative delays may accelerate the progression of lesions that might otherwise have been identified and treated at an early stage.

At the same time, the pandemic has highlighted the need for additional diagnostic technologies—such as capsule endoscopy and telemedicine. However, the rapid implementation of these innovations has been uneven, and they have not yet fully replaced conventional digestive endoscopy [77,78]. It remains to be seen whether the lack of rigorous monitoring of patients with precancerous lesions will translate into a noticeable increase in advanced-stage disease in the coming years, and whether effective strategies can be implemented to recover the losses incurred during the pandemic.

### 6.3. Disruption of Oncologic Management

The pandemic has posed significant challenges to oncologic management, and in the case of gastric cancer—an already high-risk malignancy—the consequences have been particularly stark. Elective and urgent surgical interventions, such as gastrectomies, have frequently been postponed or rescheduled due to the reallocation of operating rooms and anesthesiology staff to COVID-19 units, resulting in delays that could negatively affect long-term outcomes [38,52,53]. Even when surgeries have proceeded, the preoperative process has required complex protocols for testing and isolation, leading to extended waiting times and increased hospitalization costs.

Similarly, chemotherapy and radiotherapy have faced additional obstacles related to limited outpatient scheduling, restricted access to dedicated oncology spaces, and reduced staff availability. Quantitatively, a large systematic review and meta-analysis (245 studies, 46 countries) reported an overall 28% decline in cancer treatment delivery during the pandemic, with modality-specific reductions ranging from 15% for radiotherapy to 35% for systemic treatment (including chemotherapy) compared with pre-pandemic periods [79].

Service-level radiotherapy data also demonstrate measurable disruptions. Using the National Radiotherapy Dataset in England, mean weekly radiotherapy courses decreased by 19.9% in April 2020 versus April 2019 (with attendances/fractions dropping by 29.1%), and reductions persisted in May (courses −6.2%; attendances −31.4%) and June (courses −11.6%; attendances −31.5%) [80]. To reduce hospital visits, hypofractionation was adopted more frequently; for example, ultra-hypofractionated breast regimens (26 Gy in 5 fractions) increased from 0.2% of courses in April 2019 to 60.6% in April 2020 [80].

In upper gastrointestinal oncology, registry-based data from the Netherlands further suggest shifts in multimodal treatment patterns during the first pandemic year, including a reduction in the proportion of potentially curable esophagogastric cancer patients treated with resection and neoadjuvant chemoradiotherapy (35.0% in 2017–2019 vs. 27.3% in 2020) [81]. These challenges have sometimes necessitated modifications to treatment regimens—favoring less toxic protocols or alternatives that reduce hospital visit frequency (e.g., oral therapies or hypofractionated radiotherapy)—although the long-term efficacy of these adjustments remains to be fully evaluated [73,74,75]. For patients with advanced gastric cancer, the need for prompt, multimodal treatment has often been in conflict with the risks of viral exposure and the limited capacity of oncology centers to maintain their standard patient throughput. The pandemic has exposed profound vulnerabilities in healthcare infrastructures and underscored the importance of inter-specialty collaboration, digitalization, and robust emergency planning to prevent critical disruptions in diagnosis and treatment.

To consolidate the quantitative findings that are discussed across Section 4.1, Section 6.1, and Section 6.3 (endoscopy volume decline, stage migration, treatment disruptions, and early recovery signals), we added two summary tables: Table 1 maps the key numerical indicators to the main supporting studies, and Table 2 provides a pre-/during-/post-COVID comparative overview.

**Table 1:** Key quantitative indicators reported for endoscopy volume decline, stage migration, treatment disruption, and screening recovery in gastric cancer during COVID-19 (selected studies). Indicators and time windows differ across studies; values are therefore reported as described by each source and are not intended for direct cross-country comparison.

Domain	Setting/Data Source (as Described in Text)	Comparison Window	Key Quantitative Signal(s)	Ref.
Screening participation	South Korea (screening survey data)	2019 vs. 2020	Screening rate 70.8% → 68.9%; screening within past year 32.7% → 27.2%	[51]
Screening/claims-based signals	South Korea (claims-based analysis)	2019 vs. 2020	Esophagogastroduodenoscopies −6.3% (2020 vs. 2019); advanced gastric cancer claims −3.6%	[50]
Endoscopy volume decline	Multiple centers/multi-region reports	Peak pandemic months vs. baseline	60–80% decline in elective endoscopies	[47,48,49]
Endoscopy volume decline + early recovery	UK National Endoscopy Database (NED)	Nadir vs. May 2020	Endoscopic activity ~5% of pre-pandemic at nadir; ~20% by May 2020 after catch-up initiatives	[58]
Endoscopy volume decline	US GIQuIC registry	Mar–Sep 2020 vs. baseline	−ar–Sep 2020 vs. baselineoscopy	[59]
Stage migration/early-stage detection	Hiroshima (Japan)	Early COVID period vs. baseline	Early stage diagnoses decreased (e.g., stage I −23.9%)	[52]
Diagnostic volume + stage shift	South Korea (Yonsei Cancer Center cohort)	2018–2019 vs. 2020–2021	Diagnoses 6336 → 4539 (264 → 189/month; −28.4%), with higher proportion of advanced-stage disease	[55]
Diagnostic rate decline	Italy/other Europe + United States	Pandemic period vs. baseline	Diagnostic rates fell up to −15.9% (Italy/Europe); estimated annual decline ~−8.9% (US)	[46,48]
Treatment disruption (overall)	Meta-analysis across oncology care	Pandemic vs. pre-pandemic	Overall treatment delivery decline ~28%; radiotherapy ~15%; systemic therapy ~35%	[79]
Radiotherapy service impact	England (National Radiotherapy Dataset)	April 2020 vs. baseline	Mean weekly radiotherapy courses −19.9%; attendances −29.1%	[80]
Multimodal treatment pattern change	Netherlands registry-based data (upper GI oncology)	2017–2019 vs. 2020	Potentially curable esophagogastric cancer treated with resection + neoadjuvant chemoradiotherapy 35.0% → 27.3%	[81]
Screening recovery (system-level)	Systems with established screening infrastructure	Post-acute phase	Faster recovery reported; near-complete recovery to pre-pandemic screening rates within one year (South Korea)	[50,52]

**Table 2:** Comparative overview of key gastric cancer pathway indicators before, during, and after the acute COVID-19 disruption (selected examples).

Care Pathway Domain	Pre-Pandemic Baseline (Illustrative)	During COVID-19 Disruption	Early Recovery/Post-Acute Phase
Screening participation/claims-based screening signals	In South Korea, screening participation was high pre-pandemic (e.g., overall screening rate 70.8% in 2019, and screening within the past year 32.7%) [51].	In 2020, screening indicators declined (e.g., overall screening rate 68.9%, screening within the past year 27.2%) [51]. Claims-based signals also changed (e.g., esophagogastroduodenoscopies decreased 6.3% in 2020 vs. 2019; advanced gastric cancer claims decreased 3.6%) [50].	Regions with established screening infrastructure tended to recover faster; South Korea showed near-complete recovery to pre-pandemic screening rates within one year [50].
Diagnostic endoscopy volume	Routine elective endoscopy capacity and organized endoscopy workflows were generally stable and supported early detection.	Many centers reported 60–80% declines in elective endoscopy during peak months [47,48,49]. UK NED activity fell to ~5% of pre-pandemic at the nadir, rebounding to ~20% by May 2020 after catch-up initiatives [58]. US GIQuIC: −33.4% EGD and −38.5% colonoscopy (Mar–Sep 2020) [59].	Catch-up and prioritization approaches partially restored capacity (e.g., NED rebound by May 2020) [58]. Faster recovery was reported where screening systems were already established [50,52].
Stage at diagnosis/diagnostic rates	Earlier-stage detection is clinically critical (stage-dependent outcomes are markedly different) [56,57].	Stage migration signals were reported: in Hiroshima (Japan) early-stage diagnoses fell (e.g., stage I −23.9%) [52]. In a Korean single-center cohort (Yonsei), diagnoses decreased (6336 → 4539; −28.4%) with a higher proportion of advanced-stage disease [55]. Other settings showed diagnostic reductions (Italy up to −15.9%, US approx. −8.9%) [46,48].	Where diagnostic volumes recovered more rapidly, this may have partially offset downstream harm [50,52]. (However, endpoints and follow-up remain heterogeneous across studies.)
Surgery/operative pathways	Curative-intent surgery and perioperative pathways were generally delivered on standard schedules.	Surgical interventions were frequently postponed/rescheduled due to resource reallocation and staffing constraints [38,52,53].	Gradual restoration occurred as services recovered, but the pace differed by health-system capacity and pandemic waves (described across included reports) [38,46,54].
Systemic therapy and radiotherapy delivery	Standard multimodal therapy (perioperative chemotherapy and/or chemoradiotherapy where indicated) remained the norm.	Across oncology, a meta-analysis reported an overall 28% decline in treatment delivery during the pandemic, with modality-specific reductions (~15% radiotherapy, ~35% systemic therapy) [79]. In England, radiotherapy courses fell (e.g., −19.9% in April 2020) with fewer attendances (−29.1%) [80]. Registry data from the Netherlands suggested shifts in multimodal treatment patterns (e.g., resection + neoadjuvant chemoradiotherapy 35.0% in 2017–2019 vs. 27.3% in 2020) [81].	Many centers used regimen adaptations (e.g., less toxic/oral options, hypofractionation) to reduce visits, while longer-term oncologic impact remains under evaluation [73,74,75].

## 7. COVID-19 Vaccination and Gastric Cancer

### 7.1. Effects of Vaccination on Oncology Patients

Widespread vaccination against SARS-CoV-2 has become a cornerstone of global efforts to control the pandemic, significantly reducing the risk of severe COVID-19 and its associated mortality [82,83]. In patients with gastric cancer, the benefits of vaccination have been demonstrated by a lower likelihood of developing serious respiratory and systemic complications, as well as by enabling the continuation or timely resumption of oncologic treatments under safer conditions. Vaccination has been shown to decrease the incidence of severe disease, which in turn allows hospitals to manage oncology cases more efficiently and maintain access to essential diagnostic procedures such as endoscopy and imaging for proper cancer staging [84,85].

Although the immune response in oncology patients may be somewhat diminished compared to the general population, studies indicate that most patients with solid tumors—including those with gastric cancer—develop protective antibody levels post-vaccination, even if these titers are lower than in individuals without malignancies [85,86]. This partial immunization is generally sufficient to offer significant protection, reducing both the frequency and severity of infections while supporting a more continuous delivery of oncologic therapies. In certain cases, booster doses are recommended for patients whose initial immune response is inadequate—particularly if they are undergoing chemotherapy or immunotherapy, which can alter their immune profile [82,87,88].

Vaccination has also alleviated anxiety and reduced patient hesitancy about hospital visits, where the perceived risk of infection has previously deterred individuals from undergoing endoscopic, surgical, or systemic treatments [89]. This is especially critical for patients with intermediate or advanced gastric cancer, where timely treatment initiation can markedly influence prognosis. By providing a safer environment with a lower COVID-19 risk, vaccination helps ensure adherence to chemotherapy schedules and minimizes preoperative delays. This strategy facilitates clearer treatment planning and reduces uncertainties related to the cancelation of procedures during potential surges in infections [90,91].

### 7.2. Implications for Endoscopic and Oncologic Practice

As vaccination campaigns have expanded among healthcare personnel and patients, many endoscopy units and surgical departments have been able to relax some of the initial restrictions and streamline diagnostic and treatment workflows [49,88,89]. This has enabled the resumption of gastric cancer screening programs—not only in regions where such programs existed prior to the pandemic but also in areas that have more recently introduced systematic endoscopic diagnosis. Vaccination of medical teams has notably reduced concerns regarding viral transmission during aerosol-generating endoscopic procedures, which were previously considered high risk for spreading the virus [49,62].

Furthermore, vaccinated patients have gained greater confidence in the safety of hospital environments, as evidenced by increased consultation rates and a higher number of scheduled endoscopies and other gastric investigations [58,59]. In several countries, the gradual easing of strict protocols has also involved reorganizing patient pathways so that COVID-19 and non-COVID-19 areas are managed separately. This reallocation of resources has shortened waiting times and allowed for an increased volume of procedures, including the reinstatement of elective surgeries for gastric cancer that had been temporarily postponed [71,72].

Nevertheless, challenges remain regarding suboptimal immune responses in certain patients with advanced gastric cancer—especially those receiving immunosuppressive treatments. In such instances, clinical guidelines recommend monitoring antibody levels and adjusting treatment plans accordingly, either by administering additional vaccine doses or by implementing enhanced protective measures in the hospital setting [85,86]. In addition, ongoing patient education remains crucial to ensure that individuals understand the benefits of vaccination, its potential side effects, and the importance of resuming oncologic procedures. The emergence of new viral variants, which may impact vaccine effectiveness, underscores the need for flexibility and continuous monitoring in the management of patients with gastric cancer [82,84,88].

Overall, the widespread rollout of COVID-19 vaccines has been a pivotal step in safely reopening medical services, reducing morbidity and mortality among cancer patients, and enabling clinics and hospitals to better manage oncologic caseloads. In the context of gastric cancer—where early diagnosis and prompt treatment directly correlate with improved outcomes—the restoration of comprehensive screening and complex treatment protocols has been greatly facilitated by vaccination efforts and the implementation of safety protocols for both patients and healthcare providers.

## 8. Diagnosis and Therapeutic Approaches in Gastric Cancer Within the Pandemic Context

### 8.1. Endoscopic and Imaging Techniques

Health safety restrictions and the risks associated with aerosol-generating procedures have necessitated significant modifications in the conduct of endoscopic examinations and imaging studies required for the diagnosis and staging of gastric cancer. In numerous centers, mandatory pre-procedural COVID-19 testing and the use of high-level personal protective equipment (PPE) have become standard practices to safeguard both healthcare staff and patients [49,62]. These measures have led to more stringent scheduling and a reduced procedural capacity, forcing physicians to prioritize urgent cases or those with a high suspicion of advanced gastric cancer. Additionally, patient pathways have been reorganized to clearly separate those with positive COVID-19 tests from those testing negative, thereby minimizing the risk of nosocomial transmission [49,71].

For imaging services, reduced availability was driven predominantly by operational constraints (resource reallocation, workflow adjustments, and scheduling backlogs) rather than imaging-specific procedural transmission risk. [49,62,71]

To overcome the limitations imposed on conventional endoscopy, alternative or supplementary imaging modalities have increasingly been utilized. Computed tomography (CT), magnetic resonance imaging (MRI), and even PET/CT have been employed for staging and post-treatment monitoring, offering the advantage of systemic evaluation without direct contact with the gastric mucosa [92,93]. However, these methods generally do not match the diagnostic accuracy of endoscopy combined with biopsy for early gastric lesions, raising challenges in differential diagnosis and in ruling out other mimicking conditions. While innovations such as magnetic capsule endoscopy have shown promise, their widespread adoption has been limited by equipment availability, high costs, and the requisite level of expertise [77,78]. Telemedicine and virtual consultations have also been integrated as tools for remotely interpreting imaging results, reducing the need for in-person visits while providing ongoing monitoring for patients with known gastric lesions that do not require immediate intervention [64,78].

However, despite their benefits, these emerging technologies also present limitations, including reduced diagnostic accuracy compared to traditional endoscopy, cost-related barriers, and variability in clinician expertise, thus necessitating careful consideration when integrating them into standardized diagnostic workflows [64,77,78,92,93].

### 8.2. Therapeutic Management

The pandemic has posed major challenges in maintaining continuity in oncologic therapies, and gastric cancer—often an aggressive malignancy—has placed additional pressures on multidisciplinary teams. Minimally invasive endoscopic procedures, such as EMR and ESD, have been prioritized for patients with early lesions and high risk of progression, as these interventions offer high curative potential and typically require shorter hospital stays [20,21,22]. Nevertheless, diagnostic delays and backlogs limited timely access for some patients, increasing the likelihood that disease would be detected at a more advanced stage [54].

Regarding surgical management, many units have been forced to delay scheduled gastrectomies, particularly during peak pandemic periods when intensive care beds and surgical staff were redirected to COVID-19 care [75,94,95]. In cases where emergency surgery was unavoidable (such as for massive hemorrhage, perforation, or obstruction), physicians operated under strict isolation protocols, and the risk of perioperative complications was elevated due to reduced staffing and constrained postoperative care conditions. Chemotherapy and radiotherapy regimens have similarly been adapted—often shifting to less toxic protocols, reducing the number of hospital visits, or employing hypofractionated radiotherapy—in an effort to minimize patient exposure to healthcare settings [73,74]. Simultaneously, telemedicine has provided a framework for managing adverse treatment reactions and monitoring stable patients, allowing for timely adjustments to treatment plans without frequent hospital visits [64,78]. However, careful evaluation is needed to assess the long-term effectiveness of these modifications, as gastric cancer generally demands intensive, multidisciplinary management. Some of the adapted treatment regimens may not sustain the optimal therapeutic outcomes, underscoring the importance of clear crisis-management guidelines to ensure that oncology patients are not disadvantaged by competing medical emergencies [71,72,74].

### 8.3. Considerations Regarding the Safety of Medical Teams

Since endoscopic procedures and certain surgical interventions for gastric cancer are considered aerosol-generating, the healthcare teams performing these procedures are at high risk for SARS-CoV-2 exposure. The use of advanced PPE (including FFP2/FFP3 masks, face shields, and waterproof gowns) as well as negative-pressure rooms has become standard practice in many centers. However, strict adherence to these measures has inevitably slowed patient throughput due to additional preparation and disinfection time [49,62,96]. Regular COVID-19 testing of healthcare staff in high-volume endoscopy units has also been crucial for the rapid detection and isolation of asymptomatic cases, thereby preventing nosocomial outbreaks.

The vaccination of medical personnel has further enhanced safety, significantly reducing the incidence of severe disease among healthcare workers and enabling more organized operation of surgical blocks and endoscopy suites [88,90]. Despite this, variations in vaccination rates and the emergence of new viral strains with differing transmissibility have necessitated continued vigilance, meaning that stringent protective measures remain in place even after most staff have been immunized. Thus, ensuring the safety of medical teams is a multifaceted challenge that is essential not only for protecting patients but also for sustaining uninterrupted oncologic care during a public health crisis.

## 9. Discussion

The COVID-19 pandemic has profoundly altered the landscape of gastric cancer diagnosis and treatment, exposing long-standing weaknesses in healthcare systems while simultaneously accelerating the adoption of innovative practices. This duality—of crisis-driven disruption and rapid innovation—offers valuable insights into the resilience of oncologic care. By synthesizing the available evidence, several recurring themes and critical gaps emerge that should inform future preparedness strategies.

### 9.1. Synthesis of Common Themes

Because most included studies were observational, the relationships described here should be interpreted as associations and temporal trends rather than confirmed causal effects. Across global cohorts, pandemic-related restrictions and the pervasive fear of nosocomial SARS-CoV-2 infection led to sharp declines in elective endoscopies and routine gastroenterological consultations [46,49,50,51]. This reduction in procedural volume was consistently associated with diagnostic delays across multiple cohorts, with gastric cancer more often identified at advanced stages and potentially poorer outcomes. Yet, the rapid deployment of telemedicine platforms, the introduction of pre-procedural testing, and the widespread use of enhanced PPE provided a framework that allowed many regions to resume specialized oncologic services, albeit at reduced capacity [49,90]. As illustrated in Figure 1, COVID-19 has affected gastric cancer management on multiple levels—from reduced elective endoscopies and diagnostic delays to treatment adaptations and increased reliance on telemedicine.

### 9.2. Critical Comparative Analysis

#### 9.2.1. Regional Differences and Therapeutic Strategies

Countries with mature screening infrastructures, notably Japan and South Korea, demonstrated relative resilience by implementing catch-up strategies and leveraging robust national healthcare systems [52,54]. In contrast, European and North American centers adopted structured prioritization and triage protocols, with the UK’s National Endoscopy Database (NED) showing a rebound in activity from 5% to 20% within two months of lockdown [58], while the US GIQuIC registry quantified a one-third decline in both EGD and colonoscopy, guiding phased service recovery [59]. Conversely, low-resource settings faced a disproportionate reallocation of staff and infrastructure toward COVID-19 units, resulting in longer delays, higher rates of emergency presentations, and increased late-stage diagnoses [38,47]. These disparities emphasize the importance of context-specific resilience strategies and highlight how structural inequalities exacerbate oncologic outcomes during crises.

#### 9.2.2. Limitations of Alternative Approaches

Although capsule endoscopy, telemedicine, and virtual consultations provided innovative stopgaps during the pandemic, their long-term utility remains uncertain. While these tools increased accessibility and reduced exposure risks, questions persist regarding their diagnostic precision for early-stage lesions and their cost-effectiveness compared to conventional endoscopy [77,78]. Such limitations underscore the need for rigorous comparative validation before integrating these modalities into standard gastric cancer pathways.

### 9.3. Integrating an Innovative Perspective

#### 9.3.1. Toward a Conceptual Model for Oncologic Resilience

The pandemic has revealed the urgent need for a conceptual model of oncologic resilience in gastric cancer care. Across the studies reviewed, resilience emerged not as a single intervention, but as the capacity of health systems to preserve timely diagnosis and treatment, protect patients and staff, and learn rapidly from evolving evidence despite major disruption. To translate this into a practical framework, the elements described in previous sections can be organized into four interrelated, measurable domains: (1) patient safety and infection control; (2) diagnostic and treatment continuity; (3) digital and telemedicine integration; and (4) system management and monitoring.

The patient safety and infection control domain encompasses strategies that maintain access to oncologic care while minimizing infectious risk, such as standardized pre-procedural triage and testing, COVID-19 vaccination programs targeting vulnerable cancer populations, re-design of endoscopy and surgical pathways to reduce exposure, and clear communication of safety protocols to patients and staff [71,72,97,98]. In practice, this domain can be monitored through indicators such as nosocomial SARS-CoV-2 and other healthcare-associated infection rates, vaccination coverage among patients and healthcare workers, adherence to personal protective equipment and testing policies, and the proportion of procedures performed in designated “cold” or low-risk pathways.

The diagnostic and treatment continuity domain reflects the ability to sustain essential cancer services despite resource constraints. In gastric cancer this includes maintaining urgent endoscopy and staging procedures, safeguarding access to curative surgery and systemic therapy, and implementing prioritization algorithms that distinguish truly deferrable from non-deferrable care [72,78]. Relevant indicators include recovery of diagnostic and screening endoscopy volumes compared with pre-pandemic baselines, time intervals from symptom onset or referral to endoscopy and to gastrectomy, changes in stage distribution at diagnosis, rates of treatment interruption or modification, and the backlog clearance time after acute waves.

The digital and telemedicine integration domain captures how effectively health systems use digital tools to support continuity of care. As illustrated in the broader oncology literature, teleconsultations, remote follow-up, electronic triage pathways, and emerging technologies such as capsule endoscopy and AI-assisted imaging can help preserve access while reducing unnecessary hospital visits [98,99,100,101]. This domain can be assessed through metrics such as the proportion of outpatient visits delivered via telemedicine, use of remote pre-assessment pathways before endoscopy or surgery, implementation of structured digital symptom-tracking tools, and validation of alternative diagnostic strategies (e.g., capsule-based or AI-supported workflows) against conventional endoscopy.

Finally, the system management and monitoring domain refers to the data infrastructure and governance mechanisms that allow continuous situational awareness and rapid adaptation. Key components include national or regional registries for gastric cancer and COVID-19, real-time dashboards tracking procedure volumes and waiting times, multidisciplinary decision structures for reallocating resources, and mechanisms for international collaboration and benchmarking [71,72,99,102,103]. Indicators in this domain include the existence and coverage of population-based registries, the timeliness of data reporting, the use of regular audit cycles to adjust priorities, and participation in multicenter or international data-sharing initiatives.

Taken together, these four domains provide an operational scaffold that can be tailored to different health-system contexts. Rather than proposing a rigid scoring system, the model offers a set of measurable dimensions and example indicators that policymakers, clinicians, and hospital managers can use to audit oncologic resilience, identify vulnerabilities, and design targeted interventions before and during future crises.

In this review, ‘resilience’ is used conceptually to describe service capacity to absorb disruption and recover core diagnostic and treatment pathways. Because the included studies reported non-uniform endpoints, we did not apply a single predefined KPI set; however, resilience could be operationalized using service-level indicators such as endoscopy volume recovery, backlog clearance/time-to-diagnosis, stage shift, time-to-treatment, treatment completion, and cancelation/no-show rates.

#### 9.3.2. Future Directions

Looking forward, crisis-specific guidelines must evolve to integrate digital tools, adaptive surgical strategies, and personalized oncology care. These frameworks should not only aim to restore pre-pandemic diagnostic volumes but also proactively recover the “lost cases” that went undiagnosed during the crisis through intensified post-pandemic screening programs [104,105,106]. Prospective studies are also needed to clarify the long-term effects of diagnostic delays, treatment interruptions, and pandemic-related lifestyle changes on gastric cancer progression. Only through such comprehensive recovery strategies can oncology systems mitigate the pandemic’s collateral damage and emerge stronger for future global health challenges.

### 9.4. Identification of Knowledge Gaps

Despite significant progress, critical knowledge gaps remain. First, the comparative precision and scalability of capsule endoscopy versus conventional endoscopy remain insufficiently validated, particularly for early gastric lesions [77]. Second, the long-term impact of pandemic-induced lifestyle changes—including sedentary behavior, poor diet, and heightened psychosocial stress—on the evolution of preneoplastic conditions requires longitudinal analysis. Third, the role of AI-based diagnostic platforms in resource-limited environments remains underexplored, though these technologies hold promise for democratizing access to high-quality oncologic care [101]. Finally, the immunologic interplay between SARS-CoV-2 infection, systemic inflammation, and the gastric tumor microenvironment is poorly understood and warrants mechanistic investigation [25,29,31]. Addressing these gaps will require multinational collaborations, prospective data collection, and the integration of digital health and AI-driven approaches into routine clinical workflows.

### 9.5. Concluding Perspective

Ultimately, the COVID-19 pandemic should be viewed not only as a disruptive event but also as a catalyst for reimagining gastric cancer care. By fostering adaptive resilience, embracing technological innovation, and investing in international collaboration, the oncology community can transform lessons from this crisis into sustainable strategies that protect vulnerable populations against future global health threats.

### 9.6. Limitations

This narrative review supported by a structured literature search is limited by the heterogeneity of the included evidence (study designs, outcomes, and settings), the English-language restriction, and reliance on observational data, which precludes causal inference. We did not perform a meta-analysis or compute pooled effect sizes, and some service-level indicators were reported inconsistently across studies, limiting direct comparability.

## 10. Conclusions

The COVID-19 pandemic has profoundly disrupted gastric cancer care, leading to delayed diagnoses, reduced access to screening, and interruptions in surgical and systemic treatments. At the same time, it has accelerated the adoption of new practices—telemedicine, infection-control protocols, and minimally invasive interventions—that may shape future standards of care.

What distinguishes this review is its proposal of a conceptual model of oncologic resilience **structured into four measurable domains—patient safety and infection control, diagnostic and treatment continuity, digital and telemedicine integration, and system management and monitoring—**which combines structured triage, digital technologies, international registries, and harmonized safety measures into a durable framework for crisis preparedness. This integrative perspective moves beyond documenting the pandemic’s damage, offering instead a strategy to transform short-term adaptations into long-term improvements in gastric cancer management.

Future priorities must include recovering undiagnosed cases through intensified screening, validating innovative tools such as capsule endoscopy and AI-based diagnostics, and addressing the long-term impact of lifestyle changes induced by the pandemic. Strengthening international collaborations will be essential to ensure equity, rapid knowledge transfer, and coordinated responses in future global health crises.

Thus, while COVID-19 exposed vulnerabilities in oncologic care, it also provided the momentum to reimagine gastric cancer management—shifting from reactive measures toward a more resilient, patient-centered, and future-ready paradigm.

## Data Availability

The original contributions presented in the study are included in the article/Supplementary Material, further inquiries can be directed to the corresponding author.

## Supplementary Materials

The following supporting information can be downloaded at: https://www.mdpi.com/article/10.3390/life16010161/s1, File S1: PRISMA 2020 Checklist (location of items in the manuscript).
