# Supplementary material for: Learning from an Emerging Infection: How the COVID-19 Pandemic Reshaped Gastric Cancer Care
Source: Life (Basel). 2026 Jan 19;16(1):161. doi: 10.3390/life16010161 (PMC12842618; doi:10.3390/life16010161)
Supplement: Supplementary file 1 [file life-16-00161-s001.zip › life-3918444-supplementary.pdf]

**Supplementary File S1. PRISMA 2020 Checklist (location of items in the manuscript)**

Manuscript: life-3918444 — "Learning from an Emerging Infection: How the COVID-19 Pandemic Reshaped Gastric Cancer Care"

Note: Items marked "N/A" are not applicable because the review uses a narrative/scoping synthesis without meta-analysis.

| Item | Section | Checklist item (summary) | Where reported in manuscript | Notes |
|---|---|---|---|---|
| **1** | Title | Identify the report as a systematic review/scoping review | Title page (update title to include 'Scoping Review') | Planned edit |
| **2** | Abstract | Structured abstract (objectives, methods, results, conclusions) | Abstract | Reported; add counts/timeframe if needed |
| **3** | Introduction | Rationale | Section 1.1–1.2 | Reported |
| **4** | Introduction | Objectives/questions | Section 1.3 | Reported |
| **5** | Methods | Eligibility criteria | Section 2.4 | Reported |
| **6** | Methods | Information sources (databases, dates) | Section 2.2 | Reported (last search date included) |
| **7** | Methods | Search strategy (full strings/limits) | Section 2.3 | Reported; consider adding exact Scopus/WoS strings |
| **8** | Methods | Selection process (screening, reviewers, disagreements) | Section 2.5 | Reported (2 reviewers, consensus) |
| **9** | Methods | Data collection process | Section 2.7 | Reported (standardized form; cross-check) |
| **10** | Methods | Data items extracted | Section 2.7 | Reported |
| **11** | Methods | Risk of bias/quality assessment | Section 2.8 | Reported (JBI-adapted; empirical only) |
| **12** | Methods | Effect measures | N/A | No meta-analysis / effect measures not pre-specified |
| **13a** | Methods | Synthesis approach and how studies were grouped | Section 2.7 + Tables 1–2 | Narrative synthesis |
| **13b** | Methods | Data preparation/handling | Section 2.7 | Narrative; no transformations |
| **13c** | Methods | Methods used to present results | Tables 1–2; Sections 4–8 | Reported |
| **13d** | Methods | Methods to synthesize results | Section 2.7 | Narrative synthesis; no pooling |
| **13e** | Methods | Exploration of heterogeneity | N/A | Not applicable (no meta-analysis) |
| **13f** | Methods | Sensitivity analyses | N/A | Not applicable |
| **14** | Methods | Reporting bias assessment | N/A | Not performed |

| | | | | |
|---|---|---|---|---|
| **15** | Methods | Certainty assessment (e.g., GRADE) | N/A | Not performed |
| **16a** | Results | Study selection (numbers at each stage) | Figure 2 + Section 2.5 | Reported |
| **16b** | Results | Excluded studies and reasons | Figure 2 + Section 2.5 | Reported (low relevance; insufficient data) |
| **17** | Results | Study characteristics | Figure 3; Tables 1–2; Sections 4–8 | Reported |
| **18** | Results | Risk of bias within studies | Section 2.8 (qualitative) | No separate RoB table |
| **19** | Results | Results of individual studies | Tables 1–2; Sections 4.1, 4.2, 6.1, 6.3 | Reported (selected quantitative examples) |
| **20a** | Results | Summary of findings across studies | Section 9.1–9.3 + Tables 1–2 | Reported |
| **20b** | Results | Investigations of differences (subgroups) | Section 9.2 | Qualitative regional comparisons |
| **21** | Results | Reporting biases | N/A | Not assessed |
| **22** | Results | Certainty of evidence | N/A | Not assessed |
| **23** | Discussion | Interpretation, limitations, implications | Section 9 | Reported |
| **24** | Other | Registration/protocol | Section 2.1 | Protocol not registered |
| **25** | Other | Support/funding | Funding section | Reported |
| **26** | Other | Competing interests | Conflicts of Interest section | Reported |
| **27** | Other | Data/materials availability | Data Availability Statement | Reported (Not applicable) |
